# First report of root-knot nematodes (*Meloidogyne* species) infecting Chinese Elm (*Ulmus parvifolia*) in Florida, USA

**DOI:** 10.21307/jofnem-2020-049

**Published:** 2020-05-21

**Authors:** M. R. Moore, J. A. Brito, S. Qiu, C. G. Roberts, L. A. Combee

**Affiliations:** 1 Molecular Diagnostics Laboratory, Florida Department of Agriculture and Consumer Services, Division of Plant Industry, Gainesville, FL, 32608; 2 Nematode Diagnostic Laboratory, Florida Department of Agriculture and Consumer Services, Division of Plant Industry, Gainesville, FL, 32608

**Keywords:** Chinese Elm tree, Guava root-knot nematode, *Meloidogyne arenaria*, *Meloidogyne enterolobii*, *Meloidogyne javanica*, Pacara earpod tree root-knot nematode, Regulatory, Ulmaceae, *Ulmus parvifolia*

## Abstract

Samples of galled roots, resembling those induced by root-knot nematodes, and rhizosphere soil were collected from potted plants of *Ulmus parvifolia* cvs. Allee and Drake in Lake County, Florida. Nematode species were identified using both molecular analysis and morphology of perineal patterns. *Meloidogyne enterolobii* and *M*. *javanica* were identified from *U*. *parvifolia* cv. Allee. *Meloidogyne arenaria* and *M. javanica* were identified from *U*. *parvifolia* cv. Drake. This is a first report of these nematode species infecting Chinese Elm in Florida.

Chinese Elms (*Ulmus parvifolia* Jacq.: Ulmaceae: Rosales) are valued for their tough lumber, resistance to some elm pests and pathogens (Mittempergher and Santini, 2004; Bosu and Wagner, 2007), and as hardy, urban ornamentals. Native to East and Southeast Asia, *U*. *parvifolia* has an extensive cultivation history that resulted in many recognized cultivars (e.g., Santamour and Bentz, 1995). In 2019, samples of soil and roots were collected from potted plants of *U*. *parvifolia* cvs. Allee (Fig. 1A, B) and Drake (Fig. 1A), in Lake County, FL and submitted for nematode certification at the Florida Department of Agriculture and Consumer Services, Division of Plant Industry, Gainesville, FL (FDACS-DPI). Initially, nematodes were extracted from soil and roots, and species identification performed using FDACS-DPI’s standard protocol for identifying *Meloidogyne enterolobii* Yang and Eisenback, 1981, a COI-based qPCR assay (Kiewnick et al., 2015; Braun-Kiewnick et al., 2016) with slight modifications (Moore et al., 2020).

**Figure 1:**
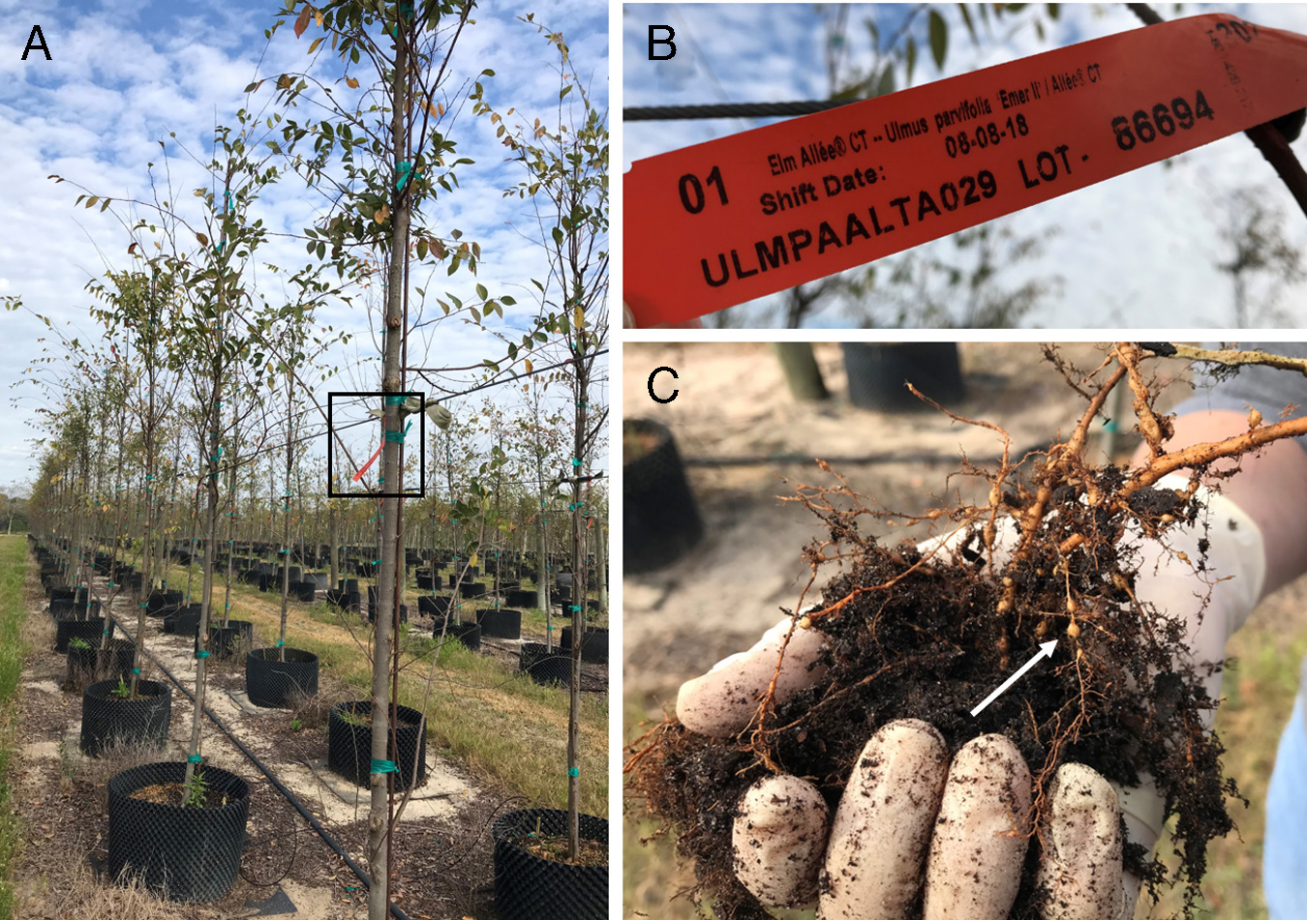
(A) Rows of Chinese Elm, *Ulmus parvifolia* Jacq. cvs. Allee and Drake in Lake County, Florida. Black square highlights the Allee cultivar tag; (B) *Ulmus parvifolia* cv. Allee tag; (C) Root galls on *Ulmus parvifolia* cv. Allee. White arrow indicates the distinctive round galls commonly produced by *Meloidogyne enterolobii* Yang and Eisenback, 1981.

These initial screening tests revealed the presence of *M*. *enterolobii* in some, but not all, soil samples collected from *U*. *parvifolia*. To determine whether *U*. *parvifolia* is indeed a host of *Meloidogyne* species including *M*. *enterolobii*, rather than weeds growing together in the pots with these elms, additional soil and root samples (*n* = 3) were collected directly from the rhizosphere of *U*. *parvifolia*. These samples were designated with internal FDACS-DPI sample identifiers N20-110, N20-113 (both from *U*. *parvifolia* cv. Allee), and N20-115 (from *U*. *parvifolia* cv. Drake). Round galls, resembling those commonly induced by *M. enterolobii*, were observed on secondary and tertiary roots of *U*. *parvifolia* cv. Allee in one of the samples (Fig. 1C), whereas in the other *U*. *parvifolia* samples, the root galls were less rounded and more like those induced by other *Meloidogyne* spp.

*Meloidogyne* species were identified using two qPCR assays (Kiewnick et al., 2015; Braun-Kiewnick et al., 2016), isozyme analyses, morphology of the perineal patterns, and concatenated NADH5/COXII sequences (Table 1). DNA was extracted from second-stage juveniles (J2) obtained from single egg masses on each root sample using the Qiagen DNeasy Blood and Tissue Kit (Qiagen^®^, Hilden, Germany) and used for qPCR, conventional PCR, and sequencing. The cytochrome c oxidase subunit I (COI) and intergenic spacer 2 (IGS2) qPCR assays were repeated with DNA from J2 extracted directly from the roots of each cultivar. Standard PCRs targeted NADH-ubiquinone oxidoreductase chain 5 (NADH5) and cytochrome c oxidase subunit II (COXII) using the primers NAD5F2/NADH5R1 and COX2F/COX2R, respectively, and thermocycle conditions described by Janssen et al. (2016). Purified PCR products were sequenced bidirectionally on an Applied Biosystems SeqStudio platform with BigDye Terminator v. 3.1 cycle sequencing chemistry (Applied Biosystems, Foster City, California).

**Table 1.** Diagnostic tests used to identify *Meloidogyne* species extracted from *Ulmus parvifolia* roots.

*Meloidogyne* isolate	Plant cultivar	COI qPCR	IGS2 qPCR	Isozyme analyses	Perineal pattern	NADH5/COXII barcodes
N20-110-2B	*U. parvifolia* cv. Allee	*M. enterolobii*	*M. enterolobii*	*M. enterolobii* (*n* = 26)a	*M. enterolobii* (*n* = 17)a	*M. enterolobii*
N20-110-3B	*U. parvifolia* cv. Allee	*M. enterolobii*	*M. enterolobii*	*M. enterolobii* (*n* = 26)a	*M. enterolobii* (*n* = 17)a	*M. enterolobii*
N20-110-6B	*U. parvifolia* cv. Allee	*M. enterolobii*	*M. enterolobii*	*M. enterolobii* (*n* = 26)a	*M. enterolobii* (*n* = 17)a	*M. enterolobii*
N20-113-1B	*U. parvifolia* cv. Allee	Undetermined	Undetermined	*M. javanica* (*n* = 26)a	*M. javanica* (*n* = 11)a	*M. javanica*
N20-113-14B	*U. parvifolia* cv. Allee	Undetermined	Undetermined	*M. javanica* (*n* = 26)a	*M. javanica* (*n* = 11)a	*M. javanica*
N20-113-18B	*U. parvifolia* cv. Allee	Undetermined	Undetermined	*M. javanica* (*n* = 26)a	*M. javanica* (*n* = 11)a	*M. javanica*
N20-115-1A	*U. parvifolia* cv. Drake	Undetermined	Undetermined	*M. javanica* (*n* = 13)a	*M. javanica* (*n* = 13)a	*M. javanica*
N20-115-6A	*U. parvifolia* cv. Drake	Undetermined	Undetermined	*M. javanica* (*n* = 13)a	*M. javanica* (*n* = 13)a	*M. javanica*
N20-115-10A	*U. parvifolia* cv. Drake	Undetermined	Undetermined	*M. javanica* (*n* = 13)a	*M. javanica* (*n* = 13)a	*M. javanica*
N20-115-16B	*U. parvifolia* cv. Drake	Undetermined	Undetermined	*M. javanica* (*n* = 13)a	*M. javanica* (*n* = 13)a	*M. javanica*
N20-115-1B	*U. parvifolia* cv. Drake	Undetermined	Undetermined	*M. arenaria* (*n* = 13)a	*M. arenaria* (*n* = 13)a	*M. arenaria* + *M*. sp. n. 1 T585
N20-115-2B	*U. parvifolia* cv. Drake	Undetermined	Undetermined	*M. arenaria* (*n* = 13)a	*M. arenaria* (*n* = 13)a	*M. arenaria* + *M*. sp. n. 1 T585
N20-115-3B	*U. parvifolia* cv. Drake	Undetermined	Undetermined	*M. arenaria* (*n* = 13)a	*M. arenaria* (*n* = 13)a	*M. arenaria* + *M*. sp. n. 1 T585

**Note:** ^a^Number of nematode specimens analyzed.

Chromatograms were trimmed and assembled into sequence contigs in Sequencer 5.4.6 (Gene Codes Corporation, Ann Arbor, Michigan). Newly generated sequences (COXII: MT135524–MT135536; NADH5: MT135537–MT135546) were aligned in MEGA7 (Kumar et al., 2016) using the default settings of MUSCLE (Edgar, 2004). The new sequences were compared to the corresponding GenBank NADH5 and COXII ‘PopSets’ (PopSets: 1005137048 and 1005136704) generated by Janssen et al. (2016). Only *Meloidogyne* isolates with both NADH5 and COXII sequences were further analyzed. The alignments of NADH5 (448 bp) and COXII (323 bp) were trimmed until data were 100% complete for each terminal taxon. Alignments were then concatenated (771 total bp) and analyzed simultaneously. K2P (Kimura, 1980) distances for the concatenated dataset were calculated in MEGA 7 (Kumar et al., 2016).

A summary of identified *Meloidogyne* spp. found infecting *U*. *parvifolia* is provided (Table 1). Samples N20-110-2B, N20-110-3B, and N20-110-6B displayed Ct values ranging from 21.801 to 23.751 (mean = 22.658; *n* = 6) and were positively identified as *M*. *enterolobii* from both qPCR assays. All other samples had undetermined Ct values for the qPCR assays. COXII sequences from N20-110 samples were 100% BLASTn matches to previously published *M*. *enterolobii* data. With the concatenated matrix, isolates N20-115-1A, N20-115-6A, N20-115-10A, N20-115-16B, N20-113-1B, N20-113-14B, N20-113-18B were 100% matches to *M*. *javanica* (Treub, 1885) Chitwood, 1949 (Janssen et al., 2016 isolates A32, T429, T497, T485, T509, T520). Isolates N20-115-1B, N20-115-2B, and N20-115-3B were 100% matches to *M*. *arenaria* (Neal, 1889) Chitwood, 1949 (Janssen et al., 2016 isolates T311, T461) and *M*. sp. n. 1 (Janssen et al., 2016 isolate T585). Isozyme analyses (esterase and malate dehydrogenase) (*n* = 26 for each sample) and morphology of perineal patterns were consistent with those reported for *M. enterolobii* (VS1-S1; N1a) isolated singly from *U*. *parvifolia* cv. Allee (N20-110) and *M. javanica* (J3; N1) also found singly on this same cultivar (N20-113), and both *M. arenaria* (A2; N1) and *M. javanica* (J3; N1) identified as mixed species infecting *U*. *parvifolia* cv. Drake (N20-115). To our knowledge this is the first report of *Ulmus parvifolia* as a host of *M*. *arenaria*, *M. enterolobii*, and *M*. *javanica* in Florida.
